# Molecular Analysis of L-Asparaginases for Clarification of the Mechanism of Action and Optimization of Pharmacological Functions

**DOI:** 10.3390/pharmaceutics14030599

**Published:** 2022-03-09

**Authors:** Marina V. Pokrovskaya, Vadim S. Pokrovsky, Svetlana S. Aleksandrova, Nikolay N. Sokolov, Dmitry D. Zhdanov

**Affiliations:** 1 Institute of Biomedical Chemistry, Pogodinskaya Str. 10/8, 119121 Moscow, Russia; ivan1190@yandex.ru (M.V.P.); v-aleksandrov@yandex.ru (S.S.A.); sokolov2144@yandex.ru (N.N.S.); 2 Department of Biochemistry, Peoples’ Friendship University of Russia (RUDN University), Miklukho-Maklaya Str. 6, 117198 Moscow, Russia; v.pokrovsky@ronc.ru; 3 Laboratory of Combined Treatment, N.N. Blokhin Cancer Research Center, Kashirskoe Shosse 24, 115478 Moscow, Russia; 4 Center of Genetics and Life Sciences, Sirius University of Science and Technology, Federal Territory Sirius, Olimpiisky Prospect 1, 354340 Sochi, Russia

**Keywords:** L-asparaginase, protein engineering, substrate specificity, acute lymphoblastic leukemia, site-directed mutagenesis, immunogenicity

## Abstract

L-asparaginases (EC 3.5.1.1) are a family of enzymes that catalyze the hydrolysis of L-asparagine to L-aspartic acid and ammonia. These proteins with different biochemical, physicochemical and pharmacological properties are found in many organisms, including bacteria, fungi, algae, plants and mammals. To date, asparaginases from *E. coli* and *Dickeya dadantii* (formerly known as *Erwinia chrysanthemi*) are widely used in hematology for the treatment of lymphoblastic leukemias. However, their medical use is limited by side effects associated with the ability of these enzymes to hydrolyze L-glutamine, as well as the development of immune reactions. To solve these issues, gene-editing methods to introduce amino-acid substitutions of the enzyme are implemented. In this review, we focused on molecular analysis of the mechanism of enzyme action and to optimize the antitumor activity.

## 1. Introduction

For more than 50 years, drugs with enzymatic activity have been used in clinical medicine. Replacement therapy for pancreatic insufficiency, acceleration of wound healing or thrombolytic management are among the most successful areas of enzymatic drug use. Enzymes that irreversibly destroy certain vital amino acids are being developed as antitumor therapeutics [1]. The first bacterial enzyme introduced into routine clinical practice was L-asparaginase (L-ASNase, L-asparagine amidohydrolase (EC 3.5.1.1)) [2]. Currently, native L-ASNase from *Escherichia coli* (EcA) or *Dickeya dadantii* (formerly known as *Erwinia chrysanthemi*) (ErA), along with the pegylated form of *E. coli* asparaginase, are successfully used for the treatment of patients with acute lymphoblastic leukemia [3,4,5,6]. Normal and tumor cells require L-asparagine for their metabolic needs. Normal cells can synthesize L-asparagine for their growth with the aid of asparagine synthetase. Neoplastic cells lack the ability to synthesize asparagine due to the absence or shortage of L-asparagine synthetase and are dependent on an exogenous supply of this amino acid from the bloodstream [7]. The anticancer effect of L-ASNase is based on its ability to hydrolyze L-ASN to L-aspartate and ammonia. The exposure of tumor cells, mainly leukemic cells, to L-ASNase leads to disturbance of protein synthesis and cancer cell starvation, resulting in their death [8]. L-ASNases have been identified in mammals, birds, plants, fungi and a wide range of bacteria [9,10]. To date, dozens of microbial sources of L-ASNases have been revealed, although not all of them demonstrated cytotoxicity against leukemic cells or tumor inhibitory effects [1,10].

L-ASNases have for a long time been classified into three families: the plant type, the *Rhizobium etili* type and the bacteria type. All bacterial L-ASNases can be subdivided into two types depending on their inducibility, cellular localization, affinity to the substrate and quaternary structure [11]. Type I L-ASNases are constitutively expressed enzymes localized in the cytoplasm and have a relatively high Km for L-asparagine. L-ASNases *Bacillus subtilis* [12], *Pyrococcus horikoshii* [13] and *Acinetobacter soli* [14] are the most studied examples of type I enzymes, which show a relatively low affinity to L-asparagine, resulting in nontherapeutic applications. Type II bacterial L-ASNases are periplasmic enzymes with induced expression during anaerobiosis that have a high affinity for L-asparagine and a wide substrate specificity, resulting in potent antitumor activity [15].

The therapeutic use of L-ASNases is limited by a variety of side effects: hepato- and nephrotoxicity, dysfunctions in the central nervous system, pancreatitis, thromboembolism, mucositis, hyperglycemia and dyslipidemia [16,17,18]. Moreover, genotoxic activity was shown for L-ASNase produced by *Streptomyces ansochromogenes* [19]. Such side effects are considered to be attributed to non-specific effects of these enzymes. In addition to the well-studied antiproliferative effects of L-ASNases, which are believed to be caused by L-ASN deprivation in the tumor cell environment, several alternative mechanisms have also been suggested. Other L-ASNase substrates include L-glutamine, D-asparagine, succinic acid monoamide and asparaginyl-tRNA [20,21]. Thus, antiproliferative or side effects may appear due to their degradation. In 1970, it was shown that L-ASNase from *E. coli* may release carbohydrates from the α2-HS-glycoprotein fetuin, suggesting that hydrolysis of cell-membrane glycoproteins and inhibition of their synthesis by the enzyme can result in cell lysis [22]. This enzyme could also inhibit glycoprotein biosynthesis and lead to membrane sensitivity due to the specific effect on the concanavalin A receptor in the sensitive and resistant L5178Y murine lymphoma cell line [23]. A very surprising cytotoxic asparagine-independent mechanism was described for a *Rhodospirillum rubrum* mutant L-ASNase (RrA). RrA demonstrated regulatory capacity and could suppress telomerase activity in several human cancer cell lines, normal activated CD4^+^ T lymphocytes and xenografts of human solid tumors [24,25,26]. These observations denote the existence of complex mechanisms of action of at least one L-ASNase to a given cell line.

In this review, we summarize the results of a genetic-based molecular analysis of L-ASNases aimed at improving their pharmacological properties and clarifying their mechanisms of action.

## 2. Structures of L-ASNases and the Mechanism of Action

The practical application of L-ASNases is currently directly related to their enzymatic properties. The structural, biophysical and biochemical properties of type I and II L-ASNases have been described in experimental works [11,27,28,29,30], and detailed information about the catalytic mechanism of bacterial L-ASNases is presented in detail in the reviews by Lubkowski et al. [31] and Loch [32].

**Figure 1:**
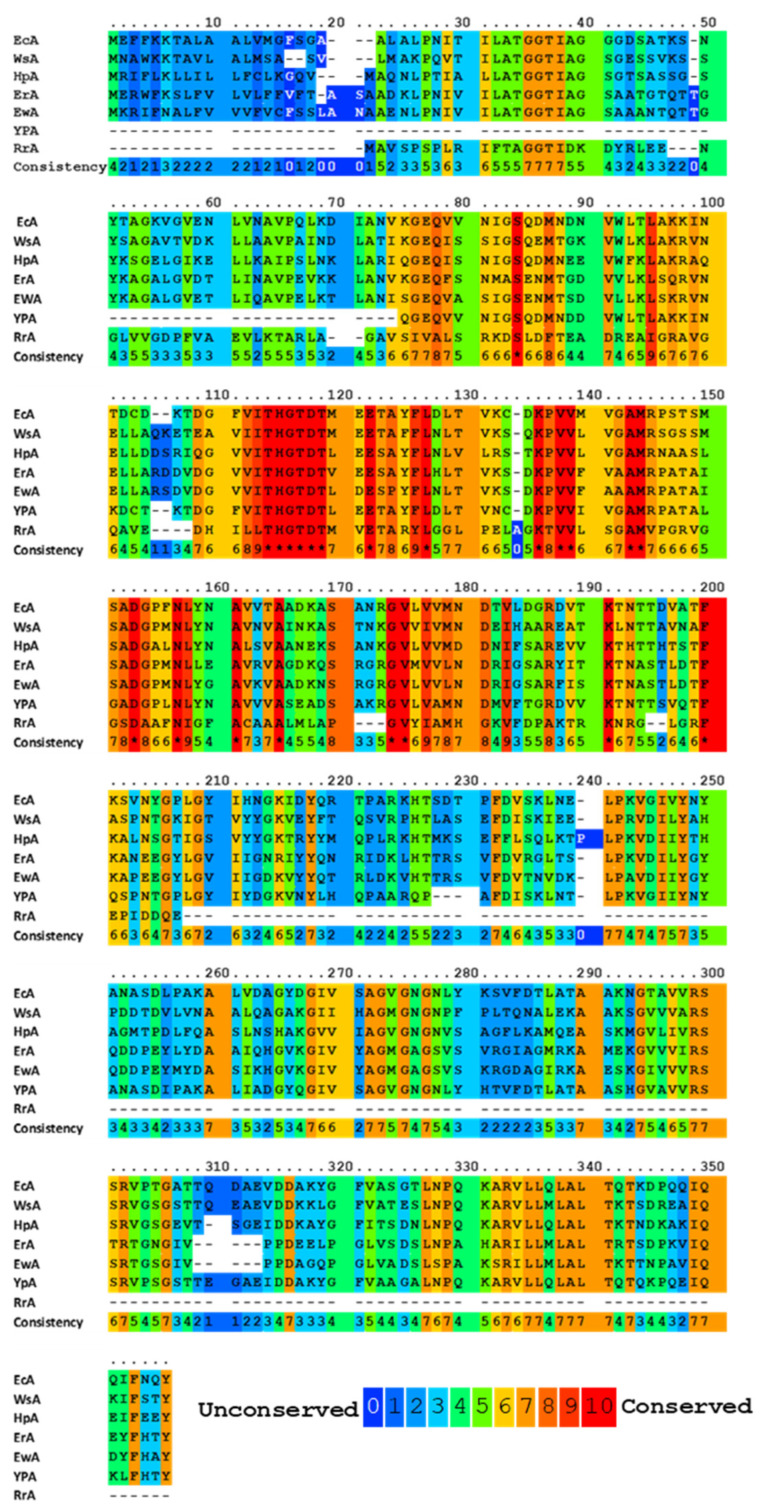
Multiple alignments and conservative residues of the most extensively studied L-ASNases. Multiple sequence-alignment toolbox PRALINE (http://ibivu.cs.vu.nl (accessed 27 May 2021) [38]) was used. Conservative residues were highlighted according to the legend. An asterisk denotes absolutely conserved residues. EcA, *Escherichia coli* II L-ASNase; ErA, *Dickeya dadantii* (*Erwinia chrysanthemi*) L-ASNase; EwA, *Erwinia carotovora* L-ASNase; HpA, *Helicobacter pylori* II L-ASNase; RrA, *Rhodospirillum rubrum* L-ASNase; WsA, *Wolinella succinogenes* L-ASNase.

All known bacterial type II L-ASNases, including those used in medicine, are highly homologous in terms of the amino acid sequence (Figure 1). Such a similarity in the amino acid sequence determines a high degree of similarity in their tertiary and quaternary structures: they fold as homotetramers with four active sites between the N- and C-terminal domains of two adjacent monomers [33,34,35,36,37]. Moreover, bacterial L-ASNases have a high degree of similarity in their tertiary and quaternary structures.

**Figure 2:**
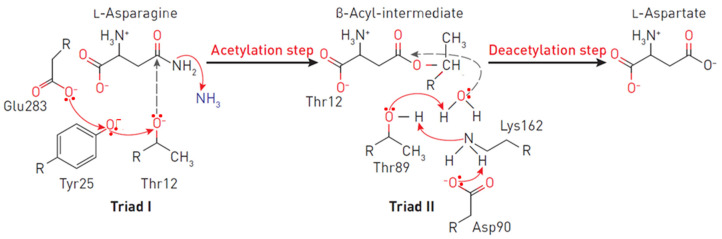
Reaction mechanism at the EcA II catalytic triads. The triad I acylates the substrate (L-asparagine) to form a β-aspartyl enzymatic intermediate. The triad II deacylates the intermediate in the presence of a water molecule to release L-aspartic acid and ammonia as products. In the first reaction, the electron density migrates from Glu283 to the oxygen of the Tyr25, and consequently, to the oxygen Thr12. A nucleophilic attack occurs, leading to the release of ammonia and the formation of ether. In the second reaction, due to the presence of a charge on Asp90, an ionic bond is formed with the amino group Lys162, leading to the removal of the proton from Thr89, followed by the nucleophilic attack of the water molecule on the carbon of the ester. Thus, a deacetylation reaction occurs.

Each monomer has 40 β-layers and 8 α-helices arranged in an N-terminal domain and a smaller C-terminal domain connected by a linker of approximately 20 amino acid residues [20,39,40]. The apex of the substrate-binding site is covered by a flexible loop (amino acid residues 14–33), which acts as a “lid”, modulating the affinity to the substrate [41,42]. One of the residues of the lid loop (in the case of EcA–N24) participates in a network of hydrogen bonds near the catalytic site (the critical residue of Y25). In all L-ASNases, the conformational change of the flexible loop after binding of the substrate creates a medium that stabilizes the negatively charged tetrahedral intermediate, thereby ensuring the effectiveness of the enzymatic reaction. From the very beginning, it was clear that catalysis occurs through nucleophilic substitution. However, the question remained: is the reaction a single substitution involving only the activated water molecule, or does it involve two stages of double substitution (also called a ping pong reaction, Figure 2) [43,44]. The mechanism of double substitution with an intermediate acylated enzyme has been challenged by experimental data on mutants T19A, T116A, T19A/T116A, K188M and Y308F of the active site of guinea pig L-ASNase in support of the mechanism of direct displacement of type I/II L-ASNases [34,45]. However, several recent papers have confirmed the original interpretation of the catalytic mechanism [20,31,46], and apparently, the exact mechanisms of enzymatic hydrolysis of asparagine of different classes differ.

**Figure 3:**
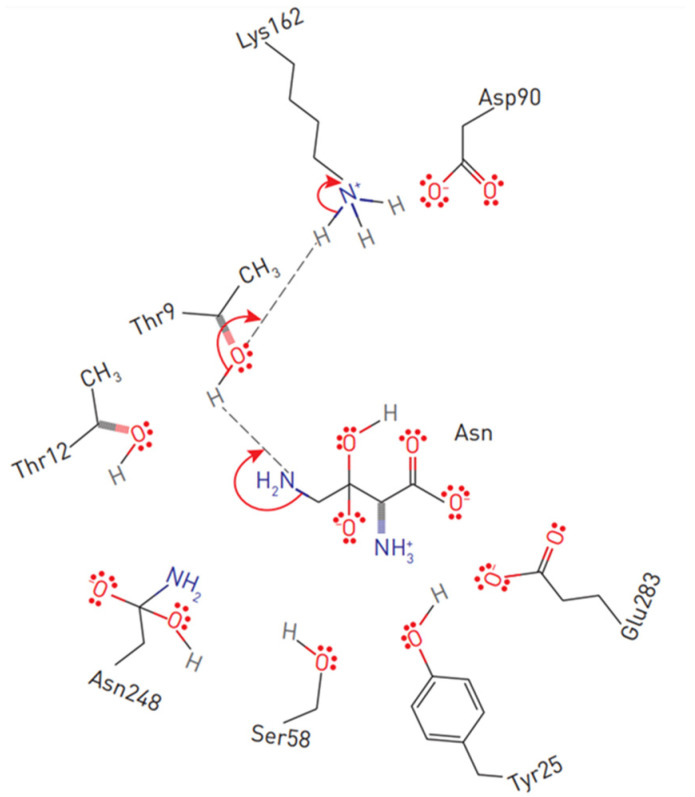
Substrate positioning in EcA II active center. Displayed are conserved active site residues with their relative locations to L-asparagine.

The primary nucleophile of the T12 enzyme was identified after the publication of the EcA structure [36,47]. After the addition of asparagine, the nucleophilic threonine, located close to the active center of the protein, interacts with the carbonyl group of the amide substrate (L-Asn) to form an intermediate product of the reaction—an acyl-enzyme. An ammonia molecule is split from the substrate to form another product of the reaction. The acyl-enzyme reacts with a water molecule to release L-ASNase and form aspartate [40,44,48,49]. Threonine activation is a combination of several coordinated effects. In the EcA enzyme, the primary role is assigned to invariant N90 as it forms a set of hydrogen bonds, which involve Y25 (Figure 3). Additional factors include the precise positioning of the substrate molecule, the participation of the water molecule, and the hydrogen bond that is formed between the nitrogen of the carboxamide group of the substrate and the carbonyl oxygen of the main chain of the enzyme in A114 [8]. The role of the catalytic activity of residues in substrate binding and specificity (T12, Y25, T89, D90 and K162 in EcA) has been established in several studies [8,50,51,52]. These residues are also conserved among other bacterial enzymes (shown in Figure 1). The coordinating role of several motifs in the first nucleophilic attack explains why “catalytically deficient” variants (i.e., lacking either T89 or Y25) may still retain residual catalytic activity. By chemical modification, 1H-nuclear magnetic resonance and 1H-NMR spectroscopy, it was determined that tyrosine and histidine are components of the active center of EcA [53].

The catalytic mechanism of L-ASNases depends on two highly conserved catalytic triads. The first consists of amino acids threonine, lysine and aspartic acid (in EcA: T12, K162 and D90), resembling serine, histidine and the aspartate triad of classical serine proteases, which mainly participate in the catalysis of the reaction. The second, consisting of threonine, tyrosine and glutamic acid (in EcA: T12, Y25 and E283), participates in the binding of the substrate and the release of reaction products [52,54,55,56]. However, another study demonstrated that E283 is not essential for catalysis [57]. 

In contrast to the triad functioning in EcA, in the ErA structure, Y29 is well ordered and forms hydrogen bound to T15, but its side chain does not interact with any residue from the second protomer in the dimer (the nearest distance to carboxylate of E289 exceeds 12 Å), which goes against the universal presence of the second catalytic triad (T, Y, E) in L-ASNases [37]. Structural analysis of ErA showed that residues T15, T95, S62, E63, D96 and A20 are in contact with the ligand [20]. The catalytic triads in L-ASNase *Helicobacter pylori* (HpA) are represented by T16, Y29, E289 and T95-D96, K168 [49]. Replacement of amino acids forming the active center of HpA T16E, T95D and T95H led to a complete loss of enzymatic activity [58]. T95 is not directly involved in catalysis, but it is involved in the activation of the water molecule responsible for the second nucleophilic attack during the deacylation step. T95 is also involved in substrate binding. Replacement of T95 with histidine or aspartate completely disrupts enzymatic catalysis.

Molecular and genetic analysis of *E. coli* genes encoding L-ASNases I and II, including 5′- and 3′-untranslated regions, revealed one region of sequence similarity and differences in 11 positions [33,59]. A typical secretory signaling peptide of 22 residues was also found, the promoter region was identified and the site of the beginning of transcription of the gene encoding L-ASNase II was determined. Its strict regulation by cyclic AMP receptor protein and anaerobiosis protein, which regulates fumarate and nitrate reductase, was confirmed.

The crystal structure of a human L-asparaginase-like protein was obtained, and by rational engineering, three double mutants and two quadruple mutants were created, which allowed the catalytic activity to be increased up to six times [60]. Structurally, the hASNase3 protein belongs to the N-terminal nucleophile (Ntn) family, which requires autocleavage between G167 and T168 to become catalytically active. To determine the individual contribution of each of the three conserved threonines of the active center (threonine triad T168, T186, T219), mutants T168S, T186V and T219A/V were prepared for enzyme-activating autocleavage reactions, and their ability to cleave and catalyze asparagine hydrolysis was tested. These studies have shown that although not all threonines of the triad are necessary for the cleavage reaction, they are all obligatory for ASNase activity.

The crystalline bacterial enzyme EcAI is organized into a tetrameric assembly, but it is not clear whether the protein exists as a tetramer in solution. The same doubt was expressed about the type I enzyme of archaea from *Pyrococcus horikoshi*, which acts as a dimer rather than a tetramer [33]. L-ASNases of some extremophile bacterial organisms [61], for example, *Thermus thermophilus* [62], *Thermococus sibiricus* [63] or *Melioribacter roseus* [64], can act as hexamers (trimers of dimers), but there is no structural evidence to support this hypothesis. It is believed that the dimer of L-ASNase II, which has two active centers, is not able to cleave L-asparagine. The effectiveness of the octamer or dodecamer is also reduced, although these functional states of the enzyme can be detected in smaller percentages in commercial preparations, which indicates an issue with the stability of the enzyme.

## 3. Stability and Activity of L-ASNases

**Figure 4:**
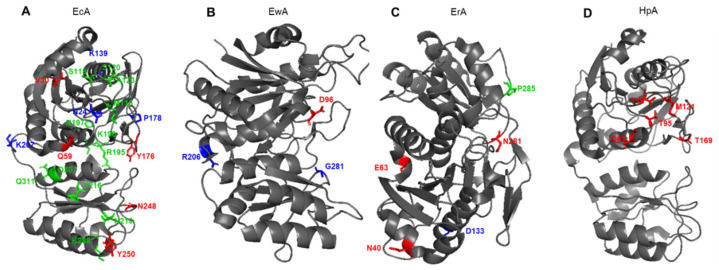
Schematic presentation of amino acid residues that determine the properties of (**A**) EcA, *Escherichia coli* L-ASNase II; (**B**) EwA, *Erwinia carotovora* (*Pectobacterium carotovorum*) L-ASNase; (**C**) ErA, *Dickeya dadantii* (*Erwinia chrysanthemi*) L-ASNase; (**D**) ErA, *Helicobacter pylori* L-ASNase. Red font shows amino acids associated with L-glutaminase activity. Blue font shows amino acids associated with the stability of the enzyme. Green font shows amino acids associated with immunogenicity.

It is generally believed that the stability and activity of enzymes are antagonistic. Several examples support this hypothesis, showing that more stable enzymes tend to exhibit a lower rate of catalyst [65,66]. Substitution E289A in HpA sharply reduced the catalytic activity of the enzyme but increased its thermal stability, which suggests a stabilizing role of such mutation [58]. In the HpA quaternary structure, E289 is located near the active site, close to Q63 from a neighboring monomer and N255 from the same monomer. Amino acids that determine the properties of the most studied L-ASNases and that are subject to molecular substitution are shown in Figure 4. The replacement of E289A in HpA led to a loss of interactions in the interunit space and with the substrate. Moreover, alanine at position 289 interrupted all interactions with N255 and Q63, which may lead to a change in the localization of the variable loop to a conformation more similar to another enzyme from *Dickeya dadantii* [20,49]. The resulting conformation could be the reason for the mutant’s inability to carry out proper catalysis.

There are several counterexamples to the rule of antagonistic relations between stability and enzymatic activity [67], and L-ASNase may be one of these exceptions. It was shown that mutations leading to an increase in the flexibility of the loop closing the substrate-binding site reduce the catalytic reaction, whereas mutations stabilizing the loop lead to an increase in the catalytic effect [68]. Variants with increased thermal stability have often improved catalytic efficiency, even if modifications are applied to subunit interfaces and their flanking residues [69,70]. Since part of the binding site is located at the monomer-monomer interface, a possible explanation is that mutations that enhance the interaction of subunits contribute to the tight binding of the substrate and improve the compactness of the tetramer. The conservative mutation A176V in the intracellular L-ASNase of *Saccharomyces cerevisiae* completely suppressed the activity of the enzyme. Sequences and structural comparisons with bacterial L-ASNases of type II have shown that the mutated residue is located in a highly conserved region and participates in the coupling of functional tetramers [71]. Verma et al. [69], using the example of several variants of EcA with amino acid substitutions, also showed that even small changes at the interface of subunits can significantly affect the stability of EcA. In a study by Vidya et al. [72], it was shown that in EcA, the most stabilizing mutations on the surface loops K139D and K207D caused neutralization and/or inversion of total protein charge and eliminated unfavorable electrostatic interactions compared to the control mutant K139R and the wild-type enzyme. In another study, the *Erwinia carotovora* variant with a single point mutation D133L had a half-inactivation temperature of 55.8 °C, whereas the wild-type enzyme had a half-inactivation temperature of 46.4 °C [73]. At 50 °C, the half-life values for wild-type enzymes and mutant enzymes were 3 and 160 h, respectively. Screening of the library of random substitutions of the D133 residue and analysis of the electrostatic potential of the wild-type enzyme showed that D133 is located in a neutral region on the surface of the enzyme and makes a significant and unfavorable electrostatic contribution to overall stability. The single-point mutation of N24S led to the creation of an EcA variant with higher thermal stability, which also retained levels of catalytic activity equivalent to the native variant and had less degradation during long-term storage [74]. The binding of L-asparagine to the allosteric site in the crystal structure of EcA I was observed simultaneously with the reorganization of the quaternary structure in this study. The carboxyl group of bound asparagine formed salt bridges and hydrogen bonds with R240, while nitrogen N (delta 2) interacted with T162. The R240A mutation increased the Km from 0.5 to 5.9 mM, presumably due to a decrease in the site’s affinity with L-asparagine. Signal transmission from the allosteric site to the active site appears to involve tiny interactions at the dimer-dimer interface and Q118 position near the active site for the binding of a water molecule.

The importance of the five tyrosine residues of EcA II for its stability and catalysis has been studied extensively by Derst et al. [52] using site-directed mutagenesis, chemical modification and thermodynamic studies of protein denaturation. While tyrosine residue Y25 is directly involved in catalysis, the hydroxyl groups of residues Y181, Y250, Y289 and Y326 were not necessary for the activity of EcA, but residues Y181 and Y326 were involved in the stabilization of the natural tetramer EcA. The pH titration curves showed that the residue of the active center Y25 has a normal pKa, while the C-end of Y326 is unusually acidic. The 1H-NMR signals of a specific ligand-sensitive tyrosine residue were assigned to Y25. None of the mutations H87A, H87L, H87K, H183L or H197L in EcA significantly affected Km in reaction with beta-hydroxamate or binding of aspartate.

The data from Wehner et al. [75] question whether histidine residues are necessary for the catalysis of EcA and suggest that H183 is important for the stabilization of the native ASNase tetramer. 1H-NMR and fluorescence spectroscopy show that H87 is located inside the protein, possibly near the active center. Single-point amino acid substitution of G281S in L-ASNase from *Erwinia carotovora* significantly affects its structural and functional properties and leads to a 10.8-fold increase in Km and a 45.5-fold decrease in catalytic activity to L-asparagine [76]. The mutant exhibited altered kinetic properties depending on pH and demonstrated an increased temperature of half-inactivation compared to the wild-type protein, which suggests that G281 contributes to the low stability of the enzyme.

Bioinformatical and structural analysis by Li et al. [77] identified structures that affect the thermal stability of thermophilic and nonthermophilic type II L-ASNases. Modification of these structures in L-ASNases from *Pyrococcus yayanosii* (PyA), *Thermococcus gammatolerans*, *Bacillus subtilis* and *E. coli* made it possible to verify that amino acids at positions 51 and 298 of PyA and correspondingly those at 57 and 305 of EcA are key amino acids responsible for the thermal stability of thermophilic and nonthermophilic type II L-ASNases. Moreover, the tightness of the C-end, the rigidity of the loop and the low surface charge near the active site are of great importance for the thermal stability of L-ASNases.

Using several modeling approaches in silico, Mahboobi et al. [78] developed a new structure of EcA to improve its pharmokinetic profile and predicted an enzyme variant with four mutations, L23G, K129L, S263C, and R291F, with lower toxicity, higher stability, and an increased half-life. L-ASNase from *Bacillus licheniformis*, when replaced by D103V, demonstrates increased thermal stability, specific activity, affinity to the substrate and a three-fold increased half-life compared to the native enzyme [79]. L-ASNase *Pyrococcus furiosus*, in the case of K274E mutation, had improved enzymatic properties under physiological conditions and showed approximately 2.5 times higher catalytic efficiency, reduced activation energy and two times less Km at 37 °C compared to the wild-type enzyme [80]. In addition, Gervais and Foote [81] found that two L-ASNase mutants from *Dickeya dadantii* have a single deamidation site, N41D or N281D, and approximately the same specific activity as the wild type. However, the double-mutant N41D N281D has increased specific activity. Structural analysis showed that small changes caused by the N41D point mutation may have reduced the number of hydrogen bonds in this α-helical part of the protein structure. The N281D mutation resulted in a decrease in glutaminase activity compared to the wild type and the N41D mutant. However, the N281D mutation also gave the enzyme less stability at elevated temperatures. In general, these data suggest that deamidation at sites N41 and N281 does not affect enzyme activity and should not cause concern during processing, storage or clinical use.

According to sequence alignment and homologous modeling, the residues of G107D, T109Q, T109S and S166A of type II L-ASNase from *Bacillus subtilis* adjacent to the catalytic cavity were replaced by site-directed mutagenesis [70]. The mutant G107D showed increased heat resistance and higher activity with L-asparagine than the wild-type protein. A comparative analysis of the interactions of hydrogen bonds, the surface electrostatic potential and the structure of the substrate-binding pocket between G107D and the wild-type protein showed that G107D replacement led to small conformational changes and redistribution of the surface electrostatic potential, while contributing to improved protein stability and catalytic efficiency.

## 4. Protease Resistance and Immunogenicity

Type II L-ASNases EcA and ErA currently used in clinical practice are characterized by instability in vivo, a short half-life [82] and the need for multiple injections to maintain a pharmacologically active concentration of enzyme in the blood [83]. In addition, L-ASNases are sensitive to the human lysosomal proteases cathepsin B (CSB) and asparagine endopeptidase (AEP), which lead to specific degradation, loss of activity and enhanced elimination [74,84,85,86]. The resistance of L-ASNases to CTSB and/or AEP affects the formation of specific antibodies and is one of the main causes of hypersensitivity reactions in patients [87]. The N24 residue on the flexible active loop in EcA was identified as the primary cleavage site for AEP. Modification of N24G at this site made EcA resistant to proteases derived from leukemia cells (AEP) and confirmed the key role of the flexible loop for enzyme activity [84]. EcA variants N24S, N24T and N24A have also increased stability and resistance to proteases [68,74,88]. The N24 residue, although not directly involved in catalysis, contributes to the preservation of asparaginase and glutaminase activity, increased antileukemic efficiency in vitro, stability during long-term storage and improved thermal parameters [74]. Structural analysis demonstrated a unique network of hydrogen bonds related to residue N24. Geometric modeling and molecular dynamics based on the normal regime predict the overall rigidity and stability of the N24G monomer compared to the wild-type monomer.

The native EcA used in clinical practice is currently being replaced by a pegylated enzyme that is more stable bloodstream [3,89,90]. Kotzia et al. [91] found that the partial proteolysis of pegylated L-ASNase from *Erwinia carotovora* by trypsin is associated with the initial cleavage of the peptide bond between K53 and G54 and then the second cleavage between R206 and S207 of the C-terminal fragment. In the study by Newsted et al. [92], it was demonstrated that L-ASNase can become resistant to trypsin by forming a chimeric protein between the enzyme and a protective single-stranded antibody. Chimeric L-ASNase retained 75% of its initial activity when exposed to trypsin, whereas native unprotected L-ASNase was completely inactivated.

The main factor of immunogenicity of L-ASNases is the presence of antigenic determinants in the sequence of the enzymes, which leads to the stable production of antibodies that reduce the therapeutic effect [93,94]. Recent reviews summarize the advances and current challenges in the deimmunization of protein therapeutics, with a special emphasis on bioinformatics tools [95,96]. The immunogenicity of protein preparations can be reduced by modifying sequences that are recognized by antibodies (B cell epitopes). Numerous B cell epitopes were identified on the surface of EcA, and several variants were created by site-directed mutagenesis [78,97,98]. The sequence change from 195RKH197 to 195AAA197 led to a noticeable decrease in the antigenicity of the enzyme. Mutations in 282GIVPPDEELP292 region led to the decrease in the immunogenicity of ErA, as well as EcA [99,100]. The binding of two hexapeptides (283IVPPDE288 and 287DEELPG292) with antibodies depended on P285 and P286 since their replacement with any other amino acid led to a decrease in binding capacity. Other residues were less important for antibody binding. Three mutant enzymes, P285T, P286Q and E288A, were expressed in *E. coli*. Kinetic characteristics of mutants P286Q and E288A for L-asparagine and L-glutamine were comparable to those for the wild-type enzyme. The Km values for the P285T mutant with both substrates were similar to those for the wild-type enzyme, whereas k_cat_ was reduced two-fold for L-asparagine and four-fold for L-glutamine. Replacing proline with threonine reduced the antigenicity of the enzyme eight-fold [99]. As a rule, a large number of mutations makes it difficult to maintain the same stability and properties of the variant in comparison to the wild-type enzyme. Ramya et al. [94] narrowed the search area to mutations that do not violate the general structure, using a phylogenetic approach to map the most conserved residues in similar epitopes of L-ASNases of several microorganisms. The authors found that tyrosine, histidine, threonine and serine make the greatest contribution to immunogenicity. Molecular modeling was used to determine the location of B- and CD4^+^ T cell epitopes of EcA or EwA, and the affinity of the produced mutant variants to the substrate was estimated. In the study by Belén et al., a structural analysis of the immunogenicity of EcA and EwA was performed, and the prediction of immunogenic and allergenic epitopes in their structure was implemented using the relative frequency of immunogenic peptides for eight alleles. This study showed that there were no significant differences in the level of immunogenicity between the two enzymes, but L-ASNase from *E. coli* had a higher relative frequency of allergenic epitopes contributing to hypersensitivity. Sequence analysis of recombinant type I L-ASNase from *Rhizobium etli* identified four conserved motifs with noticeable differences in the conserved sites of the amino acid sequences of type I and type II L-ASNases, especially in comparison with the therapeutic enzymes EcA and ErA [101,102]. These differences suggested distinct immunological specificity. When comparing the crystal structures of EcA and ErA with a computer model of type I L-ASNase, various secondary structures were revealed, and immunogenic epitopes were identified. The EcA W66Y and Y176F mutants obtained by Mehta et al. [98] were more effective against acute lymphoblastic leukemia cells than the wild-type enzyme. It has been shown that a decrease in the viability of leukemic cells occurs due to the increase in caspase-3, the release of cytochrome C, poly (ADP-ribose)-polymerase, suppression of the antiapoptotic protein Bcl-XL, cell cycle arrest in G0/G1 and ultimately, apoptosis. The variant K288S/Y176F demonstrated 10-fold reduced antigenicity in mice compared to wild-type EcA. Moreover, the serum obtained from mice immunized with wild-type EcA and from acute lymphoblastic leukemia subjected to EcA administration for several weeks recognized the K288S/Y176F mutant significantly less than wild-type EcA. Variants W66Y, Y176F and K288S/Y176F depleted asparagine and suppressed the transcription of asparagine synthetase more effectively than wild-type EcA. The use of bioinformatics, modeling and protein engineering, as well as mutagenesis methods, made it possible to create a significantly less immunogenic variant of EcA with amino acid substitutions (M115V, S118P, S120R, A123P, I215V, N219G, Q307T and Q312N). This variant maintained k_cat_ identical to the wild-type enzyme. The resulting mutant showed slightly reduced (33%) specific activity for L-glutamine and was stable in serum for more than 10 days.

The putative epitope regions of the five most studied L-ASNases YpA, Rra, WsA, EwA and EcA were obtained using Discotope bioinformatics predictions [103], ElliPro [104], EPSVR [105] and homological modeling. In experiments on mice, it was shown that all the studied L-ASNases have moderate immunogenicity with negligible cross-reactivity [106]. Antibodies produced in mice after EcA administration had the least ability to develop cross-reactivity with other enzymes. This can be explained by the low immunogenicity of EcA. Such an observation is consistent with the current position of EcA as a first-line drug for the treatment of acute lymphoblastic leukemia. Any other enzyme, preferably WsA, YpA or RrA, can be used as a second-line treatment without the risk of a significant change in pharmacokinetics due to the formation of antibodies. However, EcA may be a target for antibodies produced in mice previously immunized with any other L-ASNase due to the high immunogenicity of these proteins and cross-reacting epitopes. YpA showed the highest cross-reactivity, which can be explained by more than 74% homology between EcA and YpA sequences [107]. YpA was the most immunogenic protein compared to EcA, WsA and RrA in the mouse model. The analysis of differences associated with increased immunogenicity of tyrosine, tryptophan and charged amino acids was more conclusive than structural analysis. It is possible that the long N-end of the YpA enzyme, which is enriched with charged amino acids and tyrosine (KYIV), is the reason for the higher immunogenicity of YpA. WsA, RrA and EwA showed similar immunogenicity. Thus, the studied L-ASNases with antitumor effects can be arranged in the following order by immunogenicity: YpA > RrA, WsA ≥ EwA > EcA. Perhaps the introduction of new L-ASNases that do not have cross-reactive immunological activity to clinical practice (for example, L-ASNases from *Wolinella succinogenes* and *Helicobacter pylori*), could circumvent the issue of hypersensitivity in patients receiving multiple doses of EcA and/or ErA [108,109]. Interestingly, in the crystal structure of the EcA mutant D90E in the region involved in the immunological response, a conserved zinc-binding site was found that is formed by D100, H197 and D200 [50]. The D90E mutation mainly affected the binding of the substrate.

The immune response to large proteins is complex, and the antibodies produced are usually polyclonal, so replacing one amino acid cannot prevent the formation of antibodies against the entire modified protein. This determines the relevance of the development of new highly effective drugs of short-chain L-ASNases. Among the promising enzymes to study, RrA has half the length of amino acid sequence (172 amino acid residues) and low homology to EcA and ErA [110]. An important advantage of this enzyme is its low L-glutaminase activity (less than 0.1% of L-asparagine activity).

The immunogenicity of the protein can also be altered by sequences associated with the main histocompatibility complex II (MHC II), and they are capable of inducing T cell-dependent immune responses. There is evidence, obtained in animal models, in vitro experiments and at the early stages of clinical trials, that a violation of T cell epitopes can reduce the formation of antibodies to some therapeutic proteins [111,112]. T cell receptors on CD4^+^ T lymphocytes recognize antigenic peptides (usually 13–25 amino acids), which are present in a complex with MHC II on the surface of antigen-presenting cells. Fernandez et al. [113] and Cantor et al. [114] revealed significantly higher levels of antibodies against EcA among Caucasian patients with the HLA-DRB1 × 07 allele and three other receptors with low binding affinity. To confirm the association with acute lymphoblastic leukemia, the authors created homology models for the receptor DRB1 × 07:01 and three other receptors with low binding affinity. The free energy of binding between the epitope and DRB1 × 07:01 was significantly lower than that with the three other receptors, indicating high stability of the interaction between this peptide and MHC II. Immunization of HLA-transgenic mice expressing the leukemia-associated allele DRB1 × 0401 with the mutant protein resulted in a significant decrease in T cell responses and a 10-fold decrease in anti-EcA IgG titers compared to wild-type mice [114].

Bacterial enzymes are foreign proteins to humans. The long-term goal is to create an effective variant of human L-ASNase for use in cancer therapy without immunogenic properties [27,115,116]. However, the native form of human L-ASNase does not have therapeutic efficacy since its Km is in the millimolar range, while the concentration of asparagine in the blood is approximately 50 µmol/L [117,118,119]. For a more detailed study of biochemical and cytotoxic properties and possible modifications of human ASNase, Belviso et al. [116] expressed human ASNase in bacteria and then purified recombinant human asparaginase GST-ASPG. It has been demonstrated that the enzyme has the activity of asparaginase and PAF-acetylhydrolase, depending on the critical T19. Accordingly, ASPG, unlike the T19A mutant, exhibited cytotoxic activity in the leukemia cell lines K562, NALM-6 and MOLT-4 but not in normal cells. The study suggested ASPG as a possible antitumor agent. Another achievement was the discovery of a new human-like L-ASNase in guinea pigs with a Km in the micromolar range [28]. This enzyme was used to create a mutant variant with a 140-fold increase in catalytic activity and cytotoxic ability.

PEG modifications of L-ASNases are also aimed at reducing the immunogenicity of enzymes. This topic is excellently reviewed in recent papers [120,121,122].

## 5. Substrate Specificity

Most L-ASNases have residual glutamine activity; however, for some enzymes, it exceeds the innate asparaginase activity [123]. L-ASNases with very high glutaminase activity are grouped into the glutaminase-asparaginase family, and they have been assigned EC code 3.5.1.38. In the study by Strzelczyk et al. [46], glutaminase-asparaginase was shown to use the same ping-pong mechanism for catalysis. Attempts to test the effectiveness of *Acinetobacter glutaminasificans* glutaminase-asparaginase against acute lymphoblast leukemia in patients were discontinued due to strong side effects, presumably due to high glutaminase activity [124,125].

Despite their very similar structures, type II L-ASNases from different microbial sources exhibit different kinetics for the two substrates. For example, HpA, unlike other type II L-ASNases, has a fairly similar catalytic activity to L-asparagine and L-glutamine but an increased affinity with the first substrate by almost 150 times [126]. Aghaiypour et al. [20] obtained crystal structures of *Dickeya dadantii* L-ASNase (ErA) complexed with reaction products of L-glutamic, D-aspartic and succinic acids. These structures revealed a similar ligand-binding method; however, a comparison of four independent active sites in each complex indicates a unique and specific interaction. The larger radical side chain of L-glutamic acid than that of L-aspartic acid causes several structural distortions on the active side of ErA. The flexible loop of the active site (residues 15–33) does not demonstrate a stable conformation, which leads to a suboptimal orientation of the T15 nucleophile. The interaction of D-aspartic acid with the active site of ErA is very different from that of other ligands, suggesting that the low activity of ErA against D-aspartate can be mainly attributed to a low kcat value. Comparison of the amino acid sequence and crystal structure of ErA with those of other bacterial L-ASNases showed that the presence of two residues of the active sites E63 and S254 may correlate with significant glutaminase activity, while their replacement with Q and N, respectively, led to a decrease in glutaminase activity.

The crystal structures of ErA in combination with L-aspartic and L-glutamic acids revealed two enzyme conformations, open and closed, corresponding to the active and inactive states and explaining the preference for L-asparagine over L-glutamine [127]. Ligand binding induces the positioning of catalytic T15 into its active conformation, which allows ordering and closing of the flexible N-terminal loop. It is noteworthy that L-aspartic acid is more effective than L-glutamic acid in inducing active T15 [128]. ErA structural analysis was used to create mutants with a reduced ability to hydrolyze L-glutamine. The main role is attributed to the E63Q mutation, which prevented the correct location of L-glutamine but not L-asparagine. Replacing S254 with asparagine or glutamine increased the specificity of the enzyme but only in combination with the E63Q mutation. The A31I mutation reduced the Km value of the substrate. Notably, the ErA variant with low L-glutaminase activity retained the ability to induce cancer cell death.

Offman et al. [68] and Mehta et al. [98] used site-directed mutagenesis of EcA to create substitutions of N24A and Y250L near the active site, which practically eliminated activity against L-glutamine and retained up to 72% asparaginase activity. The double-mutant N24A Y250 L resulted in higher compactness of the tetramer with a smaller cavity of the active center than that of the native enzyme. A decrease in glutaminase activity was possible due to steric obstacles to a larger glutamine molecule. Mutant variants Y176S and Y176F also showed reduced glutaminase activity. In addition, Y176F, as mentioned above, was more effective against acute lymphoblastic leukemia cells than wild-type EcA. As predicted by genetic algorithms and molecular dynamics, designed EcA mutant variants N24T and N24A were capable of forming an active tetrameric structure. Mutations of R195S and N24A that control glutaminase activity have demonstrated their importance for cytotoxic activity [68].

In EcA, the amino acid residue N248 participates in the formation of hydrogen bonds that affect the interaction with the substrate. A selective reduction in glutaminase activity was demonstrated for EcA in the amino acid at position 248. Among the obtained EcA mutants with a selective decrease in L-glutaminase activity, the most promising was the protein with the N248A replacement located close to the substrate-binding site [57].

There is practically no glutaminase activity in the WsA enzyme [128]. Unexpectedly, it was found that the WsA variant with proline at position 121 (WsA-P121) acquired L-glutaminase activity in contrast to the wild type with a serine residue at this position, but both variants showed comparable asparaginase activities [127]. Structural analysis of WsA in complexes with L-aspartic and L-glutamic acids suggests that residue 121 affects the conformation of conserved tyrosine 27 in the catalytically important flexible N-terminal loop [129]. The authors revealed the influence of specific amino acids of an evolutionarily conserved motif on the ability of the flexible N-terminal loop to shape its active and inactive conformation, which determines the relative substrate specificity in this L-ASNase protein family. Computer modeling has shown that a selective decrease in glutaminase activity is the result of small conformational changes affecting the residues of the active center and catalytically important water molecules [130].

Other examples demonstrate that the L-glutaminase activity of L-ASNases from various microorganisms can also be modulated [131]. In silico, Ln et al. [132] found that the replacement D96A close to the ligand-binding site of the enzyme EwA reduces the activity of glutaminase by 30% and increases the activity of asparaginase by 40%. Analysis of the crystal structure of HpA revealed some specific features, mainly in the flexible loop (residues 19–46) and in loop 286–297, which may explain the specific catalytic properties of HpA [126]. Amino acid residues E289, N255 and Q63 determine the availability of the active site in EcA. Overall, structural comparison showed that HpA has a greater structural similarity with EcA than with any other L-ASNases [49]. Maggi et al. [58,133] emphasized the important role of T16 and T95 residues in both catalytic activities of HpA. One random mutant, M121C/T169 M, had preserved efficacy against L-asparagine but was unable to hydrolyze L-glutamine. Moreover, this variant did not have a cytotoxic effect on HL-60 cells. Mutant Q63E had a similar catalytic efficiency for L-asparagine and halved L-glutaminase activity in comparison to the wild-type enzyme that leads to the potent cytotoxic activity.

Unlike wild-type EcA, the Q59 L mutant variant, which retains L-asparaginase activity but shows undetectable glutaminase activity, was inactive against cancer cells expressing asparagine synthetase (ASNS). However, this mutant protein was active against asparagine synthetase-negative cells [134]. Attempts were made to combine L-ASNases with inhibitors capable of reducing the synthesis of asparagine synthetase (for example, asparagine synthetase mRNA inhibitors), which resulted in an increase in the efficiency of removing asparagine from the blood [135]. Obviously, the lack of asparagine synthetase expression or activity in tumor cells can be considered a biological marker that allows for predicting the effectiveness of treatment [136].

## 6. Alternative Approaches to the Development of Antitumor L-ASNases with Improved Functions

The most obvious way of producing novel L-ASNases for cancer therapy is the isolation of enzymes from novel sources and evaluation of their alternative activities. In this regard, the review by Ali et al. [137] discusses studies aimed at finding useful L-ASNases from alternative sources, including bacteria, fungi, actinomycetes, algae and plants. The role of chemical modifications and protein engineering in improving the effectiveness of medicines is also discussed. Currently, as an alternative to bacterial L-ASNases with chemotherapeutic use, several type II bacterial enzymes are under consideration: *Thermus thermophiles*, *Proteus vulgaris*, *Pseudomonas fluorescens*, *Serratia marcescens*, *Erwinia aroidea*, *Aspergillus terreus*, *Mycobacterium tuberculosis*, *Yersinia pseudotuberculosis*, recombinant *Saccharomyces cerevisiae*, *Halomonas elongata*, *Sarocladium strictum*, *Streptomyces rochei*, *Aspergillus terreus*, *Fusarium culmorum* and *Zymomonas mobilis* [62,138,139,140,141,142]. They all have antiproliferative activities toward cancer cells. Attention is drawn to the abovementioned enzymes with reduced glutaminase activity cloned from *Erwinia carotovora* [40], *Wolinella succinogenes* [143], *Bacillus licheniformis* [144], *Helicobacter pylori* [126], *Pyrococcus furiosus* [145], *Streptomyces brollosae* [146,147] and *Rhodospirillum rubrum* [148]. 

Of interest are enzymes from the bacteria *Leucosporidium scottii*, *Anoxybacillus flavithermus*, extremophile yeast, *Bacillus antitudinis*, etc., living in high- and low-temperature and highly saline environments [80,149,150,151]. Recently L-ASNase from *Melioribacter roseus*, which belongs to the Ignavibacteriae Bacteroidetes/Chlorobi group, was purified and characterized. [64]. The enzyme demonstrated the optimal temperature at 70 °C and exhibited cytotoxicity toward tumor cell lines K562, Jurkat, LNCaP and SCOV-3 but not toward normal fibroblasts. The authors believe that thermal stability makes the protein more resistant to degradation in vitro and toxic with low enzymatic activity at 37 °C.

Zuo et al. [152] reported the cloning and expression of a new thermostable L-ASNase from *Thermococcus zilligii* that showed maximum activity at pH 8.5 and a temperature of 90 °C, the highest ever observed. A later L-ASNase from *Thermococus sibiricus* demonstrated similar thermal stability and was 86% active after 20 min of incubation at 90 °C [63]. 

L-ASNases found in *Rhizobium etli*, a nitrogen-fixing bacterium living in symbiosis with legumes, have very remote immunogenic properties for asparaginases used in medicine [101]. A search in the UniProt database showed that most of the homologs to L-ASNase *Rhizobium etli* are found in bacteria *Firmicutes* and *Proteobacteria*, and some homologs are also present in eukaryotes, including fungi *Ascomycetes*. L-ASNases from a variety of terrestrial microorganisms [153,154,155], plants [156,157] and fungi [158] have been studied; however, almost all failed to meet the strict criteria for antitumor drugs [159].

Perhaps with suitable protein engineering, alternative antileukemic agents will be created based on the structure of known enzymes. In this regard, several works described unique L-ASNases from marine organisms and proposed their applications in tumor therapy and the food industry [160,161,162].

Antitumor properties can be enhanced by the involvement of asparaginases in alternate cellular biochemical processes thanks to their moonlighting effects. By site-directed mutagenesis, active and stable mutant forms of type I short-chain cytoplasmic L-ASNase from *Rhodospirillum rubrum* (RrA) were cloned and purified. The mutants containing the substitutions E149R, V150P, F151T, E149R and V150P were capable of reducing the expression of the hTERT (human telomerase reverse transcriptase) telomerase subunit, suppressing telomerase activity in cancer cell lines and affecting the growth of xenograft tumors [24,25,163]. This observation suggests that RrA targets tumor growth by a dual mechanism involving asparagine deprivation and telomerase inhibition. Wild-type RrA and mutants with other substitutions (D60K, F61L and A64V, E67K), as well as other well-studied EcA, EwA or WsA mutants, did not show such ability. The increased telomerase maintains unlimited cell proliferation, and the ability of some RrA variants to suppress the expression of hTERT was associated with their increased toxicity.

As mentioned above, the double enzymatic activity of asparaginase and acetylhydrolase is also manifested by human L-ASNase ASPG [116]. Plant-type asparaginases (type three, class two) are present not only in plants but also in microorganisms, insects and mammals, including humans [164]. They belong to the family of Ntn hydrolases and often have dual isoaspartyl aminopeptidase/L-asparaginase activity (EC 3.5.1.1; EC: 3.4.19.5). The review by Nunes et al. described modern strategies for overcoming pharmacological issues of L-asparaginases, such as their immobilization on polymer-carriers and covalent attachment to nanomaterials. In this regard, immunogenicity can be partially eliminated by PEGylation of the enzyme [165], fusion with polypeptides (for example, XEN or PAS) and reductive methylation, glycosylation or polysialation [93]. After such modifications, open sites of L-ASNases that are not directly involved in substrate binding are blocked, and the availability of the protein molecule for immune system cells and proteolytic enzymes is hindered [166,167].

In the work by Sukhoverkov and Kudryashova [168], a new approach was proposed for regulating the catalytic properties of medically significant L-ASNases. This is based on the formation of covalent conjugates of the enzyme with branched chitosan copolymers (chitoPEGylation). The effectiveness of this approach was demonstrated by the development of PEG-chitosan-conjugated RrA [169]. Varying the composition of conjugates of L-asparaginase with PEG-chitosan allowed optimization of the catalytic properties of the enzyme by changing the surface charge, molecular weight, degree of branching of the polymer and size of the formed particles.

Modified heparin-binding L-ASNase from *Wolinella succinogenes* (Was79) contains amino acid substitutions V23Q and K24T, which resist trypsin, and the N-terminal heparin-binding peptide KRKKKGKGLGKKR, which is responsible for binding to heparin and to tumor cells in vitro [129,170]. When tested on a mouse model of Fischer’s lymphadenosis L5178Y, the therapeutic efficacy of Was79 was significantly higher than that of the reference enzymes. The Was79 variant can be expressed intracellularly in *E. coli* as a less immunogenic form.

A new drug of L-ASNase enclosed in red blood cells compatible with the patient’s blood group (Eryaspase) was initially approved for clinical use [171]. Encapsulation in liposomes, as well as in erythrocytes, allows the enzyme to be hidden both from active plasma proteases capable of degrading it and from the host immune system. It was reported that no antibodies are formed to the injected foreign protein, and the drug lifetime is prolonged inside these particles, and accordingly, in the bloodstream [172]. Eryaspase is expected to be approved in the US and EU for hypersensitive ALL, as well as being evaluated in phase III clinical trials in pancreatic cancer (NCT04292743) and triple-negative breast cancer (NCT03674242). If successfully registered, Eryaspase will be the first enzyme approved for solid cancers’ treatment.

## 7. Conclusions

Theoretical studies and practical experience have made it possible to predict the most significant amino acid residues in the catalytic process and obtain enzymes with improved properties. The results of introduced mutations are presented in Table 1. Recent progress in developing programmable nucleases, such as zinc-finger nucleases (ZFNs), transcription activator-like effector nucleases (TALENs) and clustered regularly interspaced short palindromic repeat (CRISPR)–Cas-associated nucleases, brought forward gene editing from a concept to clinical practice [173]. These techniques are proven to be successful to minimize the immunogenicity of some enzymes, improve their substrate specificity or introduce new binding sites responsible for certain moonlighting functions, some of which can be beneficial for cancer patients. Lack of asparagine synthetase in some pancreatic or breast cancer cells, along with the extended half-life and improved pharmacological properties of new asparaginases, open up the opportunity to extend the therapeutic potential of new enzymes and use them for solid cancers’ treatment. Actively developing approaches for de novo protein design by deep-learning neural networks such as trRosetta [174] or AlphaFold 2 [175] become attractive for creating proteins with specified properties. These techniques should be used to create potent artificial L-asparaginases with reduced immunogenicity, low specificity to L-glutamine and increased stability in the blood.

## Figures and Tables

**Table 1:** Amino acid substitutions in L-ASNases from various sources and the obtained results.

Source	Mutations	Results Achieved
*Bacillus licheniformis*	D103V	Higher thermal stability and increased L-asparaginase activity [79]
*Bacillus subtilis*	G107D	Increased stability and asparaginase activity [70]
*Escherichia coli II*	L23G, K129L, S263C, R291F	Nontoxic, increased stability and longer half-life [78]
*Escherichia coli II*	N24T, N24A	Increased asparaginase activity and protease resistance [68]
*Escherichia coli II*	N24G	Asparagine endopeptidase-resistant; retains 45% of L-asparaginase activity [84]
*Escherichia coli II*	N24A, R195S	50% glutaminase activity and ~100% L-asparaginase activity [68]
*Escherichia coli II*	N24S	Improved thermal stability and protease resistance [74]
*Escherichia coli II*	V27T	Glutaminase activity reduction and more stable [131]
*Escherichia coli II*	N24A/Y250L	~0% glutaminase activity and ~72% L-asparaginase activity [68]
*Escherichia coli II*	Q59L	0% glutaminase activity and ~80% L-asparaginase activity [134]
*Escherichia coli II*	W66Y	Significantly more apoptosis in lymphocytes from acute lymphoblastic leukemia patients [69,98]
*Escherichia coli II*	Y176F	Glutaminase activity reduction and ~100% L-asparaginase activity [69,98] Increase in Vmax and Km for L-asparagine and beta-hydroxamate [69]
*Escherichia coli II*	Y176S	Increase in Vmax and Km for L-asparagine and beta-hydroxamate Glutaminase activity reduction and ~100% L-asparaginase activity [69,98]
*Escherichia coli II*	P178N	Retention of 90% L-asparaginase activity at 50 °C [176]
*Escherichia coli II*	R195A, K196A, H197A	Less immunogenic [97]
*Escherichia coli II*	N248A	Glutaminase activity reduction [75]
*Escherichia coli II*	N248S	Glutaminase activity reduction [130]
*Escherichia coli II*	K288S, Y176F	10-fold less immunogenic Reduction of glutaminase activity Intact asparaginase activity [69,98]
*Escherichia coli II*	K139D/K207D, K139A, K207A	Increased stability [72]
*Escherichia coli II*	M115V, S118P, S120R, A123P, I215V, N219G, Q307T, Q312N	Substantially reduced immunogenicity [114]
*Erwinia carotovora* (*Pectobacterium carotovorum*)	D96A	Decreased glutaminase activity and increased asparaginase activity [132]
*Erwinia carotovora* (*Pectobacterium carotovorum*)	R206H	Resistance to trypsin degradation and higher thermal stability [91]
*Erwinia carotovora* (*Pectobacterium carotovorum*)	G281S	Increased half-inactivation temperature; decreased catalytic activity for L-asparagine [76]
*Dickeya dadantii* (*Erwinia chrysanthemi*)	E63Q	Decreased L-glutaminase activity [177]
*Dickeya dadantii* (*Erwinia chrysanthemi*)	D133V, D133L	Higher thermal stability [73]
*Dickeya dadantii* (*Erwinia chrysanthemi*)	P285T	Eight-fold reduced immunogenicity [99]
*Dickeya dadantii* (*Erwinia chrysanthemi*)	N41D, N281D	Increased asparaginase activity and decreased L-glutaminase activity [81]
*Helicobacter pylori*	T16D	Reduction of L-asparaginase and L-glutaminase activities [74]
*Helicobacter pylori*	Q63E	Decreased glutaminase activity [74]
*Helicobacter pylori*	T95E	Reduction of L-asparaginase and L-glutaminase activities [74]
*Helicobacter pylori*	M121C/T169M	Undetectable L-glutaminase activity [133]
*Pyrococcus furiosus*	K274E	Increased L-asparaginase activity, resistance to proteolytic digestion and no displayed glutaminase activity [80]
*Rhodospirillum rubrum*	D60K, F61L	Improvement of kinetic parameters and enzyme stabilization [178]
*Rhodospirillum rubrum*	E149R, V150P, F151T	Able to reduce the expression hTERT subunit of telomerase and suppress telomerase activity [24,25,163]
*Saccharomyces cerevisiae*	T64A, Y78A, T141A, K215A	99.9% loss of activity [179]
*Wolinella succinogenes*	V23Q, K24T	Resistance to trypsin degradation, decreased glutaminase activity and reduced immunogenicity [170]

## Data Availability

Not applicable.
